# Digital nutrition intervention in Older Americans Act programs impacts knowledge and desire to participate in virtual programming

**DOI:** 10.3389/fpubh.2026.1695528

**Published:** 2026-02-18

**Authors:** Renae C. Brown, Miranda A. Cook, Rachel Berton Weitnauer, Ciara G. Long, Shikia J. Crane, Aleta D. McLean, Laura M. Samnadda

**Affiliations:** 1 Cornell University, Ithaca, NY, United States; 2 Open Hand Atlanta, Atlanta, GA, United States

**Keywords:** congregate meal sites, group nutrition education, home-delivered meals, medical nutrition therapy, virtual programming

## Abstract

**Objective:**

The Older Americans Act of the United States is a federal law providing funding for meals and nutrition education, primarily for adults aged ≥ 60 years, to help them live independently. Nutrition education has traditionally been delivered in-person, but an evolving population has prompted a need to utilize digital programming to expand reach. The Georgians Receiving Insightful Telenutrition Services (GRITS) study was a 20-month program to provide group education and individual medical nutrition therapy (MNT) to adults using phone and online video conferencing. The objective of this study was to develop a virtual program for group and individual settings and to evaluate its effect on nutrition knowledge, confidence, and health behaviors, as well as overall feasibility and acceptability of delivering education in a virtual format.

**Method:**

Implementation and evaluation occurred from 2020–2023 within senior centers across four regions in the state of Georgia, USA. Group education and individual MNT sessions were led by a Registered Dietitian Nutritionist (RDN). Group education was delivered as standardized 30-min lessons over Zoom for 12–20 months, with length of time varying by site. Individual MNT was completed primarily over the phone and Zoom, with session duration of approximately 30-60 minutes for initial sessions and 15-30 minutes for follow-up sessions. Outcome measures were collected in both groups at baseline and post-intervention using short- and long-term qualitative surveys to assess program effects and acceptability of the program. Senior center staff also completed post-intervention qualitative surveys and focus groups to evaluate feasibility of program implementation. The program structure and evaluation were guided by RE-AIM framework for creating sustainable, generalizable interventions. Mixed-effects regression models were used to examine effects of the program.

**Results:**

In group education, a total of 22 centers completed at least 12 group sessions serving 5,012 duplicated clients. Participants who completed knowledge surveys showed a modest 1.6% improvement in knowledge scores at post-session compared to pre-session (95% CI: 0.4, 2.9; *p* = 0.012, FDR-adjusted *p* = 0.012). Across all confidence, motivation, and lifestyle behaviors measured, no meaningful differences from pre to post were observed. For acceptability, 87% of group education respondents (*n* = 142/163) reported to be satisfied with the nutrition sessions overall, and 79% of group education respondents (*n* = 128/163) indicated interest in continuing education in a virtual format with online video conferencing. In individual MNT, a total of 318 virtual MNT sessions were completed with 142 unduplicated clients. Among participants with matched pre- and post-surveys (*n* = 29), quality of life, general health, and confidence in problem-solving remained stable over time, with no detectable changes after MNT. At post-survey, 87% (95% CI: 0.76, 0.94; *n* = 49/55) of post-survey respondents reported at least one behavior change after participating in MNT sessions, with the most common changes being adjusting intake of foods, adjusting portion sizes, and incorporating more physical activity into their routines. Among participants who responded, 91% (95% CI: 0.80, 0.96; *n* = 49/54) reported that they found benefit in talking with the dietitian. In terms of feasibility, senior center staff reported positive feedback regarding the virtual format and its ability to expand client access to registered dietitians. Drivers of engagement for participants were novel education topics, lessons that were 30 min in length, and gift cards offered to maintain engagement. Challenges of program implementation included limited capacity of senior center staff in facilitating survey administration, difficulty tracking unduplicated participant numbers, and having participants not complete pre- and post-surveys.

**Conclusion:**

Results from this study suggest virtual nutrition education in group and individual settings was acceptable, feasible, and showed preliminary effects on knowledge and behavior among a subset of Older Americans Act clients. Future directions include long-term evaluation of virtual methods to deliver nutrition education to better serve community-dwelling older adults in ways that are sustainable, efficient, and effective for long-term health.

## Introduction

Maintaining a nutrient-dense diet is important as we age to prevent and mitigate the deleterious effects of chronic conditions (1). An estimated 95% of older adults (aged ≥ 60 years) have at least one chronic condition, and 79% have two or more (2). Tailored management of these conditions through diet, exercise, and medical care can mitigate disease complications, as well as prevent extended hospital stays and improve quality of life (3). Specifically, conditions such as diabetes, heart disease, and gastrointestinal disorders require individuals to follow specific dietary guidelines. Access to evidence-based nutrition information and qualified dietetic professionals is vital for individuals to navigate complex information and gain confidence in the ability to follow both medical and dietary recommendations. Despite data demonstrating the value of dietetic professionals in improving chronic disease outcomes (4–6), many older adults do not have access to a Registered Dietitian Nutritionist (RDN) due to limited coverage of nutrition services by insurance (7) and geographic barriers, particularly in rural areas (8). To address gaps in care, the Older Americans Act of 1965 designates funds for state and local agencies in the United States to provide home- and community-based services, with the goal to keep adults living independently in their homes and prevent unnecessary nursing home placement. Eligible individuals for the program are adults aged ≥ 60 years, their spouses, and individuals aged ≥ 18 years living with disabilities (9). Priority is given to clients who are most in need based on economic and social factors like income, disability, and risk of institutionalization. Older Americans Act programs include screening for social drivers of health, referral to resources, and implementation of services such as nutrition, transportation, and case management. Within nutrition services, participants receive nutrient-dense meals, nutrition education, and medical nutrition therapy delivered in group (congregate) and in-home (home-delivered) settings (10).

Community buildings, called senior centers, offer a central meeting place to deliver nutrition services. Across both rural and urban locations, initiatives have begun to better reach older adults in isolated regions, and access to virtual programming has become a critical resource to do so (11). Prior studies using digital interventions for nutrition education have shown positive impacts on healthy eating behaviors in children (aged 3–6 years) (12) and young adults (aged 18–35 years) (13), but data in older adults (aged ≥ 60 years) is lacking. A 2023 scoping review (LoBuono and Milovich) regarding technology use for nutrition health in older adults suggested nutrition education using webinars and “live” nutrition counseling have been implemented, but paucity in research exists in creation of standardized programs and ways to track outcomes from their implementation (14). This previous work also suggests that technology is beneficial in nutrition care for older adults, but gaps exist in geographic access, particularly due to inconsistent access to internet and technology equipment such as internet-supported mobile devices. Further, gaps in knowledge exist in determining which uses of digital nutrition education, if any, may be effective for improving nutrition knowledge and client adherence to dietary recommendations. The Georgians Receiving Insightful Telenutrition Services (GRITS) project sought to address this challenge by offering standardized virtual group nutrition education and individual medical nutrition therapy (MNT) sessions delivered by RDNs to Older Americans Act clients. By scaling an education program across 22 senior centers in a virtual format, this project offered a novel implementation of Older American Act nutrition programming. The objectives of this project were to (1) develop a virtual model for delivering group nutrition education and individual MNT with clients of the Older Americans Act and (2) evaluate its effect on nutrition knowledge, confidence, and health behaviors, as well as overall feasibility and acceptability of delivering education in a virtual format.

## Methods

### Study design

A pretest-posttest feasibility study in Older Americans Act clients aged primarily ≥ 60 years, recruited from senior centers in Georgia, was conducted between 2020 and 2023. The study created a virtual program to be delivered as group nutrition education, as well as individual MNT sessions, and used qualitative surveys to track differences in pre- and post-intervention outcomes. The project was administered by Open Hand Atlanta, a non-profit community nutrition service provider located in Atlanta, Georgia, USA. The Georgia Department of Human Services Division of Aging Services, the state unit on aging, oversaw regional Area Agencies on Aging (AAAs) which run the local senior centers. The project consisted of two arms of nutrition delivery: (1) group education sessions conducted via a cloud-based videoconferencing platform (Zoom Video Communications Inc., 2020) and (2) individual MNT sessions delivered via Zoom or by phone. Due to the nature of implementing this work in a community setting, the study structure was grounded in the RE-AIM framework, which guides how investigators design programming and evaluation to assess its applicability in “real-world” settings (15). This project was approved by the Georgia Department of Public Health Institutional Review Board (protocol ID: 211004).

### Recruitment

Initially, senior centers from three AAAs in Georgia were directly contacted and recruited to participate with their clients (Middle Georgia, Northeast Georgia, Southwest Georgia). As the program expanded, it was made available to all interested regions statewide, and an additional region (Heart of Georgia) was added. Inclusion criteria for study participants were community-dwelling OAA clients aged ≥60 years, their spouses, or individuals ≥ 18 years living with disabilities receiving congregate or home delivered meals in Georgia from 2020–2023 who were able to attend nutrition sessions independently and communicate in English. Exclusion criteria were individuals who were non-English speaking or unable to attend nutrition sessions independently due to inability to meet functional status as assessed by screening completed at OAA enrollment using the Determination of Need-Revised (DON-R) tool (16). Upon attending a nutrition program online or in the senior center, eligible individuals were educated about the GRITS program and offered the chance to participate. Interested participants completed consent forms with the assistance of senior center on-site staff. Open Hand Atlanta created the consent forms and coordinated with senior center staff for collection. Individuals who did not wish to participate in the research study were welcomed to attend group education and individual MNT sessions; they received the same programming and were not required to complete the consent forms or pre-post surveys required of the research participants. To ensure technical readiness of sites hosting the program, Open Hand staff conducted site visits as needed and provided any equipment required at no cost, which included laptops, projectors, projector screens, tablets, and mobile internet hotspot devices. Open Hand staff trained senior center staff on the Zoom platform and remained available for troubleshooting and technical assistance throughout the program duration.

### Intervention

#### Group nutrition education

The first arm of the program was group nutrition education. A standardized core curriculum of 12 nutrition lessons was created based on U. S. federal nutrition recommendations for adults aged ≥ 60 years, with special attention to topics requested by attendees of Georgia senior centers. Sessions were built to be delivered in 30 min, once per month, for 12 months. After 12 months had elapsed, participants expressed interest in additional lessons, and more were added for an expanded curriculum of 20 sessions total. Lesson topics included healthy eating on a budget, immunity, sleep health, therapeutic diets, and malnutrition. Lessons were grounded in adult learning theory, which connects learning objectives to real-world experiences, encouraging participants to apply lessons to their day-to-day lifestyle habits (17). Lesson presentations and handouts were created in Canva online graphic design software (Canva Pty Ltd., 2023).

At the time of a GRITS group education session, Older Americans Act clients gathered in-person at a senior center or joined from their home using a Zoom link provided. The majority of centers (18 of 22 centers, or 81.8%) gathered participants in the dining area of the senior center and displayed the presentation on a smart television or projected it onto a screen. A small group (2 of 22 centers, or 9.9%) provided participants with tablets to log into the group session from their own location. In some cases, multiple senior centers joined the same live session each month due to scheduling availability (10 of 22 centers, or 45%). In all cases, a senior center staff member logged onto the Zoom call to introduce the participants to the Open Hand registered dietitian who was based in Atlanta, Georgia.

On the Zoom platform, the dietitian delivered a 30-min nutrition lesson using active participation tools to ask the clients questions and keep them engaged in the online conference call. Senior center staff helped to ensure participants could hear the dietitian and facilitated questions. To end the session, participants were given a printed or digital handout with a summary of the lesson and a corresponding recipe. Participants in attendance were entered into a drawing to win a grocery store gift card, which was mailed by Open Hand Atlanta.

Once centers completed 12 monthly sessions, they were given the option to continue up to 20 sessions. Nearly half of centers (10 of 22 centers, or 45%) chose to continue past the core 12 lessons, and a small group (3 of 22 centers or, or 14%) completed all 20 sessions. For various reasons including lack of time and staff bandwidth, some centers started the group lessons but did not complete all sessions (3 centers or 14%). These 3 centers were not included in other data points.

#### Individual medical nutrition therapy

The second arm of the study, individual MNT sessions, offered an opportunity for participants to virtually meet with the RDN one-on-one to discuss nutrition needs and goals. MNT was targeted to individuals categorized as “high nutritional risk” based on the Nutrition Screening Initiative-DETERMINE Checklist (NSI-D). The NSI-D is a screening tool administered by senior centers to identify adults at nutrition risk. Conducted yearly, it asks clients questions on dietary intake, polypharmacy, social isolation, and oral health. Each question is weighted, and individuals with a score of ≥ 6 out of a maximum score of 21 are labelled high risk (18). In Georgia, these clients are mailed a letter notifying them of their score and encouraging them to follow up with a primary care provider or dietitian. As part of the GRITS intervention, the letter mailed to clients was edited to include instructions to contact Open Hand Atlanta to receive up to five sessions of MNT at no cost to the client. In addition to notifying eligible clients via mail, project staff recruited participants by posting flyers in senior centers, sending flyers with home-delivered meals, and advertising the service during group nutrition education sessions. Although individuals with high risk NSI-D scores were prioritized, MNT was available to all interested clients, regardless of NSI-D score. MNT sessions were offered via phone, Zoom, or in-person, and clients could join individually or with a care partner present.

During the individual sessions, the RDN followed principles of MNT, using an assessment, diagnosis, intervention, monitoring, and evaluation (ADIME) nutrition care process (19). The RDN paired the assessment with motivational interviewing and goal setting to facilitate implementation of the instruction provided. Client MNT notes were charted using HIPAA-compliant software (Healthie version 15.0.15, 2024).

### Data collection

Evaluation measures of the GRITS project were chosen in accordance with the RE-AIM framework, which emphasizes a focus on reach, effectiveness, adoption, fidelity of implementation, and maintenance of programs at both the system and individual level (15). Three primary methods were used to evaluate the project: surveys, observation, and focus group discussions. Documentation of evaluation measures was recorded using Microsoft Excel (Excel 2021,version 16).

#### Group nutrition education

Group education sessions were evaluated using survey tools. During the first education session, participants completed a comprehensive survey assessing demographics, diet and lifestyle behaviors, healthcare use, and confidence in managing nutritional health. After receiving 12 months of nutrition education lessons, participants recompleted this same survey with 12 additional questions to assess their satisfaction level of the program. During the program as they attended each group lesson, participants completed a 4-5-question pre-survey to assess their knowledge of the topic to be covered. Once the 30-min lesson was delivered, participants again answered the same knowledge questions found on the pre-survey, as well as 6 additional questions about their satisfaction with the service. On-site senior center staff and volunteers assisted with administration of surveys while the RDN provided instructions over Zoom. Participants who completed more than 12 group sessions continued to complete pre-post knowledge surveys at each group session but did not complete another comprehensive survey.

#### Individual medical nutrition therapy

To evaluate individual MNT sessions, a comprehensive survey was administered by an Open Hand associate via phone call or text message prior to the first MNT session. The survey assessed participant demographics, reason for attending sessions, health-related quality of life, and confidence in ability to solve problems. Once the client completed their MNT sessions, they were once again contacted via phone to complete the questions on the pre-survey, as well as additional questions assessing satisfaction, behavioral changes, health-related quality of life, and confidence in ability to solve problems.

#### Feasibility: senior center surveys and focus groups (qualitative themes)

To assess fidelity of group nutrition education intervention implementation at each group site, senior center staff were invited to participate in two complementary feedback activities: monthly surveys and end-of-program focus group discussions. Staff at each center were asked to complete a brief monthly survey to report observations, challenges, and general feedback following each group nutrition education session. At least one member of the research team was present for observation at all monthly group education sessions and recorded the number of participants for data tracking.

In July 2023, after the majority of group education sessions had concluded, senior center directors and staff from all participating or previously interested centers were invited to participate in virtual focus group discussions. Invitations were extended through email from GRITS project staff. Many directors were unable to participate due to limited staffing capacity, conflicting responsibilities, or competing program demands, which were factors frequently noted by staff as motivations for offering virtual nutrition education in the first place. Of 25 eligible centers, staff from four centers participated (16%). Focus group discussions lasted 30–60 min and were conducted over Zoom with 1–3 senior center directors per group. A semi-structured focus group discussion guide was developed to elicit feedback on program fidelity, feasibility, challenges, and facilitators for success. Questions prompted staff to reflect on their experiences implementing the virtual sessions, perceptions of participant engagement, use of technology and staffing constraints, and recommendations for future delivery. The guide aligned with evaluation objectives by directly targeting implementation fidelity (e.g., “Describe what worked well when hosting virtual sessions at your center.”), challenges (e.g., “What challenges did you experience during the program?”), and facilitators (e.g., “What supported successful participation at your center?”). Focus group discussions were recorded and detailed notes summarizing key points, illustrative examples, and contextual factors were compiled by the research team members to create thick descriptions for thematic analysis.

### Statistical analysis

All analyses were conducted using R version 4.5.2 (R Core Team, 2025).

#### Group nutrition education

Item-level knowledge scores were scored against the answer key and aggregated into session-level percent correct scores. Internal consistency of each session’s four-to-five item knowledge assessment was evaluated using the Kuder–Richardson 20 coefficient (KR-20). Higher session-level knowledge scores represent a higher percentage of correct responses (0–100%), and KR-20 values closer to 1 indicate greater internal consistency of the dichotomous knowledge items. Session-level knowledge scores were modeled using linear mixed-effects models with fixed effects for assessment timing (pre-session, post-session), number of sessions attended, participant gender, and highest level of education attained, as well as random intercepts for participants and site, consistent with established methodological guidance for evaluating changes over time (20, 21). Random slopes were not included due to the highly unbalanced repeated measures structure: participants with data available attended a mean of approximately four sessions, many attended only one or two, and attendance patterns varied substantially across individuals. Estimation of random slopes is not reliable when most individuals contribute only a small number of observations or when repeated measures do not follow a common temporal structure (20). Further, site-level slopes could not be estimated due to small and uneven cluster sizes. Accordingly, random intercept-only models provided the most stable and theoretically appropriate specification. Two models were estimated: one restricted to the core curriculum (sessions 1–12) and one including all offered sessions (sessions 1–20).

Confidence and motivation indicators were dichotomized (1 = Agree or Strongly agree, 0 = Neutral, Disagree, or Strongly disagree). Lifestyle behavior indicators were dichotomized to reflect meeting benchmarks (1 = ≥3 fruit servings/day; ≥3 vegetable servings/day; ≥5 cups water/day; ≥3 stress-relief activities/week). Pre/post changes in these outcomes were first assessed using McNemar tests. To estimate adjusted changes, logistic mixed-effects models were fit with fixed effects for time (baseline vs. follow-up), number of sessions attended, gender, education, as well as a random intercept for site to account for clustering. *p*-values were adjusted using the Benjamini–Hochberg false discovery rate (FDR) procedure to control for multiple comparisons across outcomes.

#### Individual medical nutrition therapy

For the MNT arm, pre/post matched outcomes (quality of life, general health, and confidence in problem-solving) were analyzed using paired t-tests and linear regression models adjusting for age, gender, and education. Delivery mode (Zoom vs. phone) was excluded due to insufficient variability. FDR correction was applied to the three primary adjusted tests. Post-only behavior change and satisfaction items were summarized descriptively. Full survey instruments and knowledge assessment answer keys are provided in supplementary materials; Supplementary Table 1 additionally summarizes survey instruments and variable coding.

#### Missing data

Across both program arms, all available observations were included for descriptive summaries and complete-case analysis was used for pre/post comparisons. Adjusted linear regression models were also fit using complete cases for included covariates. Session-level mixed-effects models used all available participant-session rows via maximum likelihood estimation.

Response rate varied by assessment instrument. Among group education comprehensive surveys and MNT surveys, we distinguished between all surveys returned with at least one non-missing item and surveys containing at least one analytic variable. For knowledge session assessments, percent-correct scores were only generated when at least one of the 4–5 knowledge items were completed. Based on the evaluation context, missingness was assumed to be Missing at Random (MAR), as survey nonresponse and lack of matched pre/post data primarily reflected program implementation factors (e.g., staggered rollout of survey procedures, session attendance patterns, and staff capacity) rather than participants’ unobserved outcome values. Post-only dietary change, perceived benefit, and satisfaction items were summarized using all available responses. No imputation procedures were applied.

#### Feasibility: senior center surveys and focus groups (qualitative themes)

Focus group discussion notes, audio recordings, and monthly staff survey responses were analyzed using a thematic approach. Consistent with recommended practices for ensuring credibility and trustworthiness in qualitative inquiry (22), multiple members of the research team first independently reviewed the thick descriptions and survey comments to identify salient ideas and preliminary categories to inform development of themes. The team then met iteratively to compare interpretations, discuss reflexive insights, and refine developing codes, following the collaborative and interpretative processes emphasized in reflexive thematic analysis (23). The analytic process was guided by the study’s implementation-focused aims; specifically, fidelity, challenges, and facilitators, which provided a deductive framework for initial coding while allowing inducting identification of additional context-specific themes emerging from staff experiences. Triangulation across data sources including focus group notes, direct observations of education sessions, and monthly staff feedback further supported credibility and confirmability by grounding themes in convergent evidence rather than a single data source. These iterative discussions and systematic comparisons contributed to dependability of the analytic process, ultimately producing the final themes presented in Table 1.

**Table 1:** Description of themes identified from focus group discussions with senior center staff and monthly feedback surveys completed by senior center staff.

Theme	Description
Recruitment drivers: Virtual option expanded reach of dietitian Addressing a need and “take it off the plate” of senior center staff to provide nutrition education Advertising through AAAs^1^	Staff noted that having access to a dietitian through virtual option expands reach of limited professionals and offers a service that otherwise many times the senior center staff must directly provide, describing that the service “takes it off my plate.” Many staff heard of the program offering directly through AAA presentation or indirectly from follow-up communications shared after AAA presentation.
Engagement drivers: Variety and novelty of topics Timing of presentations (shorter better than longer, late mornings) Instructor prompts and cues In-person staff available to assist Incentives	Participants are particularly engaged when a topic is novel. Short presentations keep participants engaged more easily; attention drifts if the presentation is lengthy (<30 min is ideal). Presentations offered between 10:30 a.m. and 11:30 a.m. work best due to attendee arrival and lunch times. Engagement with in-person materials (questionnaires, handouts, etc.) is stronger when instructor prompts attendees, walks them through materials, and provides examples. In-person staff at centers available to assist participants, repeat questions, etc. strengthens engagement. Engagement is also strengthened when instructor includes question prompts on-screen as well as asks verbally. Incentives drive engagement and promptness (gift card raffles), food suggested as a way to drive engagement.
Challenge: Limited capacity of staff Technology issues Participant reticence to ask questions Desire for in-person engagement	Evaluation activities such as participant tracking, survey administration assistance needs, and returning surveys were viewed as a challenge due to limited center staff capacity. Suggestions included prompting participants to complete survey questions together (guided) before and after sessions. Technology issues were common, especially internet and audio issues; trainings and one-on-one tech were helpful in resolving issues. The screens and projectors provided by the program presented challenges for some centers as some could not get the room dark enough to provide ideal visibility for the presentations. Some participants can tend to be shy to ask questions during presentation but then will ask questions to staff afterwards; at some sites, attendees are more comfortable asking questions to in-person staff and presenters – participants aware that instructor may have difficulty hearing them due to microphone placement and hesitate to engage. Attendees are accustomed to in-person presentations, and staff suggested a hybrid approach to increase comfort level of attendees.

^1^Area Agencies on Aging (AAA).

## Results

Tables 2, 3 show baseline characteristics of participants who completed the demographic questions on surveys. Participants were initially from the regions of Middle Georgia, Northeast Georgia, Southwest Georgia; when the program was expanded statewide, Heart of Georgia joined, for a total of four regions represented. The majority of participants were female, Black or African American, and with an education level of a high school diploma or less.

**Table 2:** Demographics of GRITS group education participants who completed baseline surveys.

Characteristic	Participants, count	Participants, frequency
Total	316^1^	
Mean age (range)	75 (41, 92)	
No data	11	3.5%
Gender		
Female	261	82.6%
Male	49	15.5%
No data	6	1.9%
Race* *multiple responses allowed		
Asian	0	0.0%
American Indian or Alaska Native	1	0.3%
Black or African American	193	61.1%
Hawaiian or Pacific Islander	1	0.3%
White	85	26.9%
Other	2	0.6%
Multi-racial	2	0.6%
No data	32	10.1%
Ethnicity		
Hispanic or Latino/a	4	1.3%
Non-Hispanic/Latino/a	197	62.3%
No data	115	36.4%
Education		
Less than high school degree	70	22.2%
High school or GED certificate	117	37.0%
Some college/technical school but have not graduated	60	19.0%
Two-year college or technical school	17	5.4%
Four-year college degree or more	36	11.4%
No data	16	5.1%
Annual income		
$0 - $19,268	158	50.0%
$19,269 - $25,648	40	12.7%
$25,649 - $32,028	8	2.5%
$32,029 - $38,408	5	1.6%
$38,409 and over	10	3.2%
Not sure or prefer not to answer	62	19.6%
No data	33	10.4%
Participation in funded program* *multiple answers allowed		
SNAP	89	28.2%
Medicaid	48	15.2%
Food pantry or food bank	147	46.5%
Other	3	0.9%
None	86	27.2%
No data	34	10.8%

^1^Duplicated number: participants could participate in both group education and individual MNT sessions.

**Table 3:** Demographics of GRITS individual MNT participants who completed baseline surveys.

Characteristic	Participants, count	Participants, frequency
Total	87^1^	
40–49	1	1.1%
50–59	1	1.1%
60–69	24	27.6%
70–79	30	34.5%
80–89	13	14.9%
90–99	5	5.7%
No data	13	14.9%
Gender		
Female	64	73.6%
Male	23	26.4%
No data	0	0.0%
Race* *multiple responses allowed		
Asian	0	0.0%
American Indian or Alaska Native	4	4.6%
Black or African American	63	72.4%
Hawaiian or Pacific Islander	0	0.0%
White	16	18.4%
Other	0	0.0%
Multi-racial	3	3.4%
No Data	8	9.2%
Ethnicity		
Hispanic or Latino/a	1	1.1%
Non-Hispanic/Latino/a	75	86.2%
No data	11	12.6%
Education		
Less than high school degree	14	16.1%
High school or GED certificate	25	28.7%
Some college/technical school but have not graduated	6	6.9%
Two-year college or technical school degree	9	10.3%
Four-year college degree or more	8	9.2%
No data	25	28.7%
Living alone		
Yes	49	56.3%
No	35	40.2%
Prefer not to answer	2	2.3%
No data	1	1.1%

^1^Duplicated number: participants could participate in both group education and individual MNT sessions.

### Group nutrition education: program reach

In group education, a total of 22 centers completed at least 12 group sessions serving 5,012 duplicated clients. Nearly half of centers (10 of 22 centers, or 45%) chose to continue past the core 12 lessons, and a small group (3 of 22 centers or, or 14%) completed all 20 sessions. Due to the virtual nature of this research, individual participants could not be identified among the room of clients, and thus an unduplicated participant number was not feasible. The average group session attendance was 18 participants, with a range of 3–60 participants per session. Of the 5,012 duplicated participants in group education, 316 comprehensive baseline surveys and 178 comprehensive post-intervention surveys were returned with at least one non-missing item. Of those, 280 baseline and 176 closeout surveys contained at least one analytic outcome variable, and 86 surveys could be matched pre-post.

**Figure 1:**
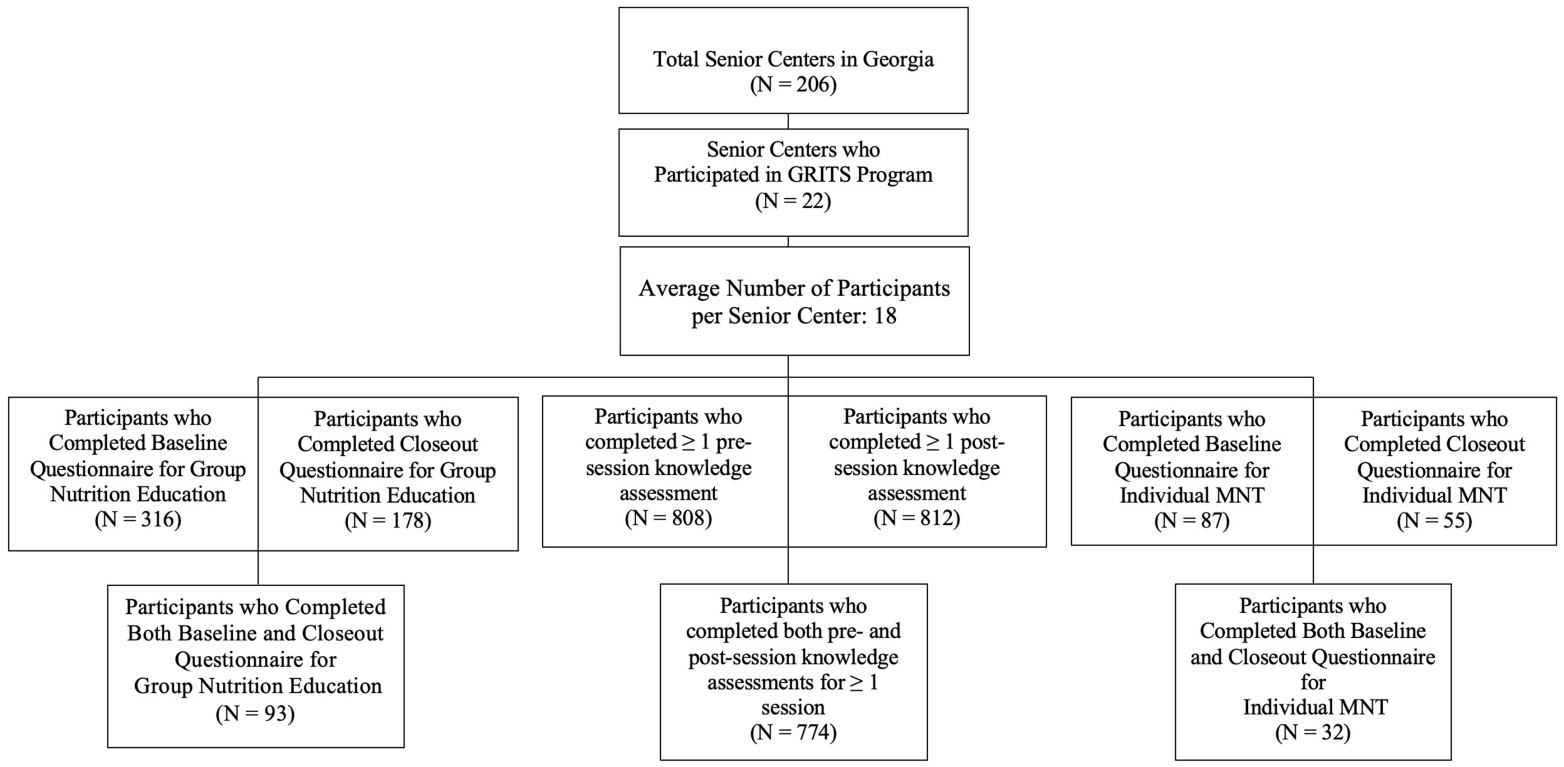
Study consort diagram.

In addition to a comprehensive survey, participants of group education completed a 4–5 question knowledge session assessment before and after each lesson. Across all sites and sessions, 3,380 pre-session and 3,336 post-session assessments were returned with at least one non-missing knowledge item, representing 808 participants who completed ≥ 1 pre-session knowledge assessment and 812 who completed ≥ 1 post-session assessment. Among individual knowledge sessions 1–20, 774 knowledge assessment surveys were able to be matched pre-post. The number of individual knowledge assessment sessions able to be matched per individual participant ranged from 1–19 with an average of 4 available per participant. A consort diagram can be found in Figure 1.

### Group nutrition education: knowledge scores

**Figure 2:**
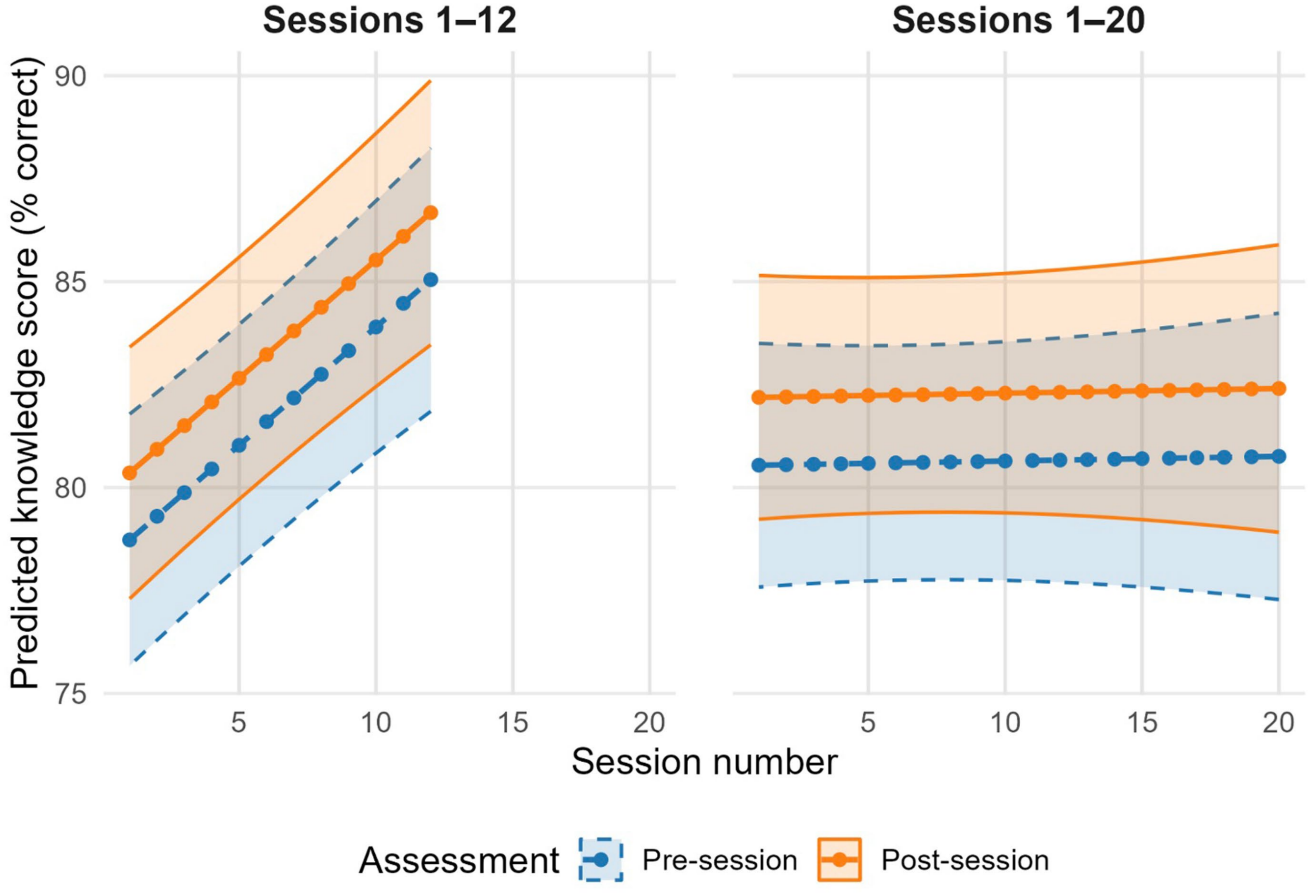
Model-based predicted pre- and post-session knowledge assessment scores for group education sessions. Points represent marginal predicted percent correct scores (with 95% confidence intervals) from linear mixed-effects models estimating session-level knowledge performance. Models include fixed effects for assessment timing (pre-session/post-session) and session number, and random intercepts for participant and site. Adjusted models include predictors: participant gender and education (reference profile: female with a high school diploma or GED). Higher scores reflect more correct responses. The left panel displays results for the primary curriculum sessions (1–12); the right panel shows results for a model including all 20 sessions delivered.

In mixed-effect models for the core group nutrition education (sessions 1–12), post-session scores were on average 1.6 percentage points higher than pre-session scores (95% CI: 0.4, 2.9; *p* = 0.012, FDR-adjusted *p* = 0.012; Table 4). In the model including all offered sessions (sessions 1–20), the estimated difference from pre to post was similar in magnitude (+1.6 percentage points, 95% CI: 0.4, 2.9; *p* = 0.008, FDR-adjusted *p* = 0.010; Table 4). For Sessions 1–12, higher session number was associated with slightly higher knowledge scores (adjusted *β* = 0.6 percentage points per session, 95% CI: 0.4, 0.8), whereas this pattern flattened when all 20 sessions were included (β = 0.0, 95% CI: −0.1, 0.2; Table 4; Figure 2).

**Table 4:** Group nutrition education: Linear mixed-effects models for session-level percent-correct knowledge scores (Sessions 1–12 and 1–20).

Effect	Sessions 1–12 *n* (participant-session-time observations)	Sessions 1–12 *β* (95% CI)	Sessions 1–12 *p*	Sessions 1–12 *p*-FDR	Sessions 1–20 *n* (participant-session-time observations)	Sessions 1–20 *β* (95% CI)	Sessions 1–20 *p*	Sessions 1–20 *p*-FDR
Post-session vs. Pre-session	3,000	1.6 (0.4, 2.9)	0.012	0.012	3,464	1.6 (0.4, 2.9)	0.008	0.016
Session number	3,000	0.6 (0.4, 0.8)	<0.001	<0.001	3,464	0.0 (−0.1, 0.2)	0.880	0.880

Coefficients (β) represent differences in percent-correct scores expressed in percentage points. Higher scores reflect more correct responses. Ns represent the number of participant-session-time observations included in each mixed-effect model. Unadjusted models include fixed effects for assessment timing (post-session vs. pre-session) and session number; adjusted models additionally include gender and highest level of education. All models include random intercepts for participant and site. *P*-values were adjusted using the Benjamini–Hochberg false discovery rate (FDR) procedure to control for multiple comparisons across outcomes.

Within group education sessions individually, standardized pre/post differences in percent correct were generally small, with Cohen’s dz. values ranging from −0.19 to 0.25, and most confidence intervals including zero, indicating that although mean knowledge scores improved slightly, the magnitude of change was modest (Supplementary Table 2). Internal consistency of the 4–5 item session-level assessments was low to moderate across sessions (KR-20 range: 0.11–0.78 pre; 0.06–0.80 post), indicating variable but generally modest reliability of the knowledge scales (Supplementary Table 3).

### Group nutrition education: confidence, motivation, lifestyle behaviors

Survey data was able to be matched pre-post for 78 participants, but due to item-level missingness, paired reporting ranges from 66 to 78 participants included. Across all confidence, motivation, and lifestyle behaviors measured, no meaningful differences from pre to post were observed. For confidence and motivation items, adjusted odds ratios for post vs. pre ranged from 0.90 to 1.71, with all 95% confidence intervals crossing 1 and FDR-adjusted *p* ≥ 0.45. For behavioral benchmarks (fruit, vegetable, and water intake; stress-relief activities), adjusted odds ratios ranged from 1.32 to 1.49, with confidence intervals including 1 and FDR-adjusted *p* ≥ 0.38 (Table 5). McNemar tests were uniformly non-significant for these domains.

**Table 5:** Group nutrition education: mixed-effects logistic models for pre/post changes in confidence, motivation, and lifestyle behaviors.

Outcome	Unadjusted *n*	Unadjusted OR (95% CI)	Unadjusted *p*	Unadjusted *p* (FDR)	Adjusted n	Adjusted OR (95% CI)	Adjusted *p* (FDR)
I have the knowledge to make healthy choices	424	1.01 (0.59, 1.72)	0.983	0.983	332	1.56 (0.71, 3.41)	0.269 (0.449)
Able to make healthy food choices on budget	432	0.62 (0.39, 1.00)	0.048	0.479	341	0.90 (0.46, 1.77)	0.766 (0.851)
Confident selecting healthy foods in grocery store	428	0.87 (0.51, 1.49)	0.62	0.775	335	1.71 (0.78, 3.76)	0.178 (0.449)
Confident cooking and preparing meals	419	0.72 (0.46, 1.15)	0.174	0.589	331	1.03 (0.52, 2.05)	0.926 (0.926)
In control of dietary/lifestyle choices	426	1.36 (0.78, 2.37)	0.277	0.589	337	1.54 (0.72, 3.29)	0.264 (0.449)
Motivated to make healthy choices	428	0.96 (0.60, 1.54)	0.869	0.966	336	1.20 (0.60, 2.41)	0.61 (0.763)
Eats ≥3 servings of fruit/day	399	1.35 (0.84, 2.18)	0.22	0.589	308	1.49 (0.78, 2.84)	0.226 (0.449)
Eats ≥3 servings of vegetables/day	410	1.20 (0.78, 1.84)	0.412	0.589	319	1.38 (0.79, 2.44)	0.26 (0.449)
Drinks ≥5 cups water/day	404	1.24 (0.82, 1.86)	0.304	0.589	314	1.38 (0.80, 2.39)	0.253 (0.449)
Stress-relief ≥3×/week	393	1.22 (0.79, 1.86)	0.368	0.589	307	1.32 (0.75, 2.33)	0.331 (0.473)

Odds ratios reflect the effect of time (post vs pre). Higher scores on confidence and motivation items indicate more positive responses. Models included random intercepts for site and are adjusted for gender and education. *P*-values were adjusted using the Benjamini–Hochberg false discovery rate (FDR) procedure to control for multiple comparisons across outcomes.

### Group nutrition education: participant satisfaction

At post, 87% of group education participants (*n* = 142/163) reported to be satisfied with the nutrition sessions overall, with 90% (*n* = 149/165) indicating the nutrition sessions provided useful information, 82% (*n* = 135/165) indicating the sessions were suited to their special needs, and 83% (*n* = 136/163) agreeing the sessions were fun and engaging. Among respondents, 91% (*n* = 149/164) reported they were glad their senior center offered the sessions, and 79% (*n* = 128/163) indicated interest in continuing the sessions in a virtual format.

### Individual medical nutrition therapy: program reach

The RDN completed 318 virtual MNT sessions with 142 unduplicated clients, resulting from 469 referrals. Of these, 87 clients returned baseline surveys with at least one demographic variable (Table 3). The average number of sessions completed was 2 out of the 5 total sessions offered. Session duration was approximately 30-60 minutes for initial sessions and 15-30 minutes for follow-up sessions, with session duration varying by client engagement and availability. All referrals were contacted and completed a session if the client was reached and expressed willingness to meet with the RDN. From tracking data, phone was the most common client preference for MNT (88% of sessions) with only a small portion participating via Zoom (12%) due to factors such as client discomfort with video conferencing and unreliable access to internet.

A total of 105 MNT clients returned at least one survey (pre-only: 50, post-only: 26, matched: 29). Of the participants who completed surveys, 79 baseline and 55 post surveys contained ≥ 1 analytic outcome variable. Survey data were almost exclusively from phone participants (*n* = 3), with no pre-post matching sets available for analysis. Matched pre-post surveys were used for complete-case analyses. All post surveys (*n* = 55) contributed to descriptive summaries of self-reported behavior change and perceived benefit.

Among participants with matched pre- and post-surveys, quality of life, general health, and confidence in problem-solving remained stable over time. Paired t-tests estimated small mean differences (quality of life: *Δ* = 0.29, *p* = 0.45; general health: Δ = 0.03, *p* = 0.85; confidence: Δ = −0.77, *p* = 0.37), with FDR-adjusted *p* ≥ 0.68 across the three tests (Table 6). In adjusted linear models with post-score as the outcome and baseline value, age, gender, and education as predictors, post-intervention scores were strongly associated with their corresponding baseline values (e.g., general health: *β* = 1.02, 95% CI: 0.48, 1.55, FDR-adjusted *p* = 0.004), as expected, while other covariates were not significantly associated with outcomes (Table 7). These findings are consistent with the paired t-tests in suggesting that, MNT component did not produce detectable changes in these measures.

**Table 6:** Medical nutrition therapy: paired *t*-test results for pre/post outcomes.

Outcome	*n*	Mean (pre)	Mean (post)	Mean change	95% CI (change)	*p*-value	FDR-adjusted *p*
Quality of life (1–7)	17	5.06	5.35	0.29	−0.51, 1.10	0.452	0.677
General health (1–5)	29	2.41	2.45	0.03	−0.32, 0.39	0.846	0.846
Problem-solving confidence (1–10)	13	7.77	7.00	−0.77	−2.57, 1.03	0.370	0.677

Ns represent the number of unique MNT participants with pre-post data for each outcome. Higher scores indicate better quality of life (1–7), better general health (1–5), or greater confidence in ability to solve problems (1–10). *P*-values were adjusted using the Benjamini–Hochberg false discovery rate (FDR) procedure to control for multiple comparisons across outcomes.

**Table 7:** Medical nutrition therapy: adjusted linear regression results for pre/post outcomes.

Outcome	*n*	*β* (per 1-point higher baseline score)	95% CI	*p* (FDR-adjusted)
Quality of life (1–7)	14	0.37	−0.17, 0.90	0.129 (0.194)
General health (1–5)	23	1.02	0.48, 1.55	0.001 (0.004)
Problem-solving confidence (1–10)	10	0.39	−3.99, 4.77	0.463 (0.463)

Coefficients (*β*) represent the adjusted association between baseline score and post-intervention scores, with higher post-intervention scores indicating better quality of life (1–7), better general health (1–5), or greater confidence in ability to solve problems (1–10). Ns reflect the number of participants with non-missing baseline, post-intervention, and covariate data for each outcome. Models adjust for participant age group, gender, and highest level of education. *P*-values were adjusted using the Benjamini–Hochberg false discovery rate (FDR) procedure to control for multiple comparisons across outcomes.

Changes assessed at post-survey only are reported descriptively. Of the 55 participants with available post-survey responses, 48 (87%; 95% CI: 0.76, 0.94) reported at least one behavior change after participating in MNT sessions, with the most common changes being adjusting intake of foods, adjusting portion sizes, and incorporating more physical activity into their routines. The majority of participants (91%; 95% CIs: 0.80, 0.96; *n* = 49) reported that they found benefit in talking with the dietitian.

### Feasibility: senior center surveys and focus groups (qualitative themes)

Three overall themes were identified from focus group discussions with senior center staff and monthly feedback surveys completed by senior center staff including: Recruitment drivers, Engagement drivers, and Challenges (Table 1). Recruitment drivers include both how senior centers learned of and became connected to the program as well as reasons staff chose to participate. Engagement drivers describe activities and aspects of programming that drove participant engagement in the program. Challenges describe the aspects of programming that made participation difficult for staff and participants.

#### Recruitment drivers

Senior center staff described learning about the program directly through presentations from GRITS program staff on AAA meetings and indirectly through follow-up communications shared after these presentations. Some staff viewed participation in the program as a way to provide senior center attendees access to a dietitian. They described having limited access to nutrition professionals for in-person programming and viewed the virtual option as a way to expand the reach of these services. Staff shared that without the program, they would typically provide the monthly nutrition lessons to their attendees, noting that not having to do so “takes it off my plate.”

#### Engagement drivers

Staff described a variety of drivers of attendee engagement including: novel topics, short presentations, providing in-person materials to complement the on-screen presentations, and instructors providing prompts for audience engagement. Staff noted that participants were particularly engaged with the sessions when the topic presented was something new and less-frequently discussed in nutrition programming. They noted that participants enjoyed the variety of topics presented. The timing and succinctness of presentations was also viewed as important, with presentations lasting 30 min or less seen as ideal. Staff noted that after 30 min of programming, attendee attention was reduced. Further, presentations held late morning between 10:30 a.m. to 11:30 a.m. worked best for the audience, as attendees tend to arrive at the centers after 10 a.m. and presentations that go past 11:30 a.m. compete with lunch and diminish engagement. Staff noted that when the instructor provided on-screen cues and prompts such as a slide with a discussion question or an activity, attendees were more likely to engage with the session. Including a printed handout or other in-person materials coinciding with the presentation also drove engagement, especially when the instructor prompted attendees to follow along with the materials. On-site staff or volunteers at the center were also key to driving engagement to help with technology, repeat questions closer to the microphone so that the instructor could hear more clearly, and assist attendees with in-person materials such as handouts and survey forms. Lastly, incentives such as a gift card raffle were viewed favorably to motivate attendees’ participation, with staff noting gift card incentives improved attendee promptness to sessions. Staff suggested snacks or other food, in addition to gift cards, as future options to encourage participation.

#### Challenges

Staff described a number of challenges faced in implementing the program including limited capacity of staff, technology issues, and for some centers, attendee reticence to engage in discussions or ask questions during the presentation. Although health literacy was not an evaluation measure, anecdotal evidence showed that both participants and senior center staff experienced difficulty logging into the virtual education platform and completing surveys in a digital format. While having on-site staff at the centers available to assist was described as an engagement driver, it was also viewed as a challenge due to the limited capacity of staff. Staff described juggling multiple competing priorities, and some centers were not able to provide as much on-site support as they would have preferred. Evaluation components of the program were seen as especially challenging for staff, as many attendees required assistance completing program surveys. Some staff also noted that tracking which participants needed to complete surveys and mailing or scanning the surveys back to program staff could be a challenge at times. Staff suggested the instructor walk participants through survey materials together as a group to help reduce the on-site staff burden and encourage completion of evaluation materials. Technology issues were common, especially related to audio and internet connection. One-on-one tech support calls offered by program staff were seen as helpful in resolving these issues. Further, flexibility and offering to reschedule sessions when internet outages occurred was beneficial. For some sites, staff shared that attendees were reluctant to ask questions or engage in discussions during the session either due to anticipated difficulty with audio (e.g., attendees felt the instructor would not be able to hear them) or a preference to discuss topics with staff members they were more familiar with. Staff suggested that a hybrid approach with occasional in-person sessions may increase attendees’ willingness to engage during the presentations.

## Discussion

Results from this study demonstrate the feasibility and acceptance of virtual nutrition education and counseling among OAA clients in Georgia, with 79% of group education respondents confirming interest in ongoing virtual format. Improvements in knowledge, confidence, motivation, and lifestyle behaviors were too modest to be educationally meaningful, but repeating the curriculum with a larger sample size of unduplicated clients may show more meaningful effects. These results are in alignment with prior studies, with a 2023 scoping review regarding digital technologies for health promotion in older adults suggesting that such programs have shown inconsistent positive outcomes (24). This and prior work support the need to ensure participants not only have access to the digital elements but also feel competent in using them. This study also supports previous intervention goals to design programs in large and simple fonts with clear categories, to provide printed hard copies in addition to digital formats, and to provide general nutrition information balanced with personalized material to suit individual participant needs. Results confirm the ongoing need to create digital content geared specifically toward older adults versus the general population (24). Additionally, it highlights the need to assess participant access to internet-enabled devices so as not to widen existing health disparities (25). Further, a 2023 survey noted that the top reasons individuals attend congregate meal programs were for socialization (26), and efforts have been made by healthcare organizations to use digital interventions to address social isolation and loneliness (27). Overall, results of this study support the potential for digital tools to improve health and healthcare delivery.

Strengths of this project included its innovative design and provision of technology supports such as projectors, projector screens, and internet-enabled tablets to senior centers and participants. Notably, its use of digital technology to connect Older Americans Act clients to an RDN, particularly in areas in which access was otherwise limited, addressed a significant gap in nutrition care access. Its coordination among a state unit on aging, a community non-profit with a team of dietitians, and a set of geographically-diverse senior centers provided a framework for future interventions. Additionally, its design of a nutrition education curriculum based on topics requested by its participants addressed a gap in prior studies. Lastly, its combination of surveys and focus groups adds evidence to a gaps in existing research; specifically, its results surrounding feasibility provide data for future digital interventions to promote health in this population.

This evaluation had several limitations. Data collection was inconsistent across senior center sites, and outcomes relied primarily on client-reported surveys, which are subject to recall and social desirability bias. In group education, unduplicated participant counts could not be collected, and survey return rates were limited across both group sessions and individual MNT; this constrained the precision with which engagement and outcomes could be quantified and reflects a common challenge in virtual programming. Knowledge assessments were intentionally brief (4–5 items per session) to reduce participant burden and support ongoing engagement. As expected for short scales, internal consistency was modest across sessions (KR-20 range: 0.11–0.78 pre; 0.06–0.80 post), and effect sizes may underestimate true learning improvements. Still, the pattern of small, positive gains was consistent across both the core curriculum (sessions 1–12) and expanded program (sessions 1–20). For the MNT component, surveys were administered primarily by phone to reduce access barriers, which may have increased social desirability bias but also enabled participation among clients with limited digital access. Although group education and MNT data used a similar identifier convention (initials and last four digits of phone number), identifiers were entered as free text and were not standardized across data sources. Formatting inconsistencies, entry errors, and lack of a validated unique identifier precluded reliable linkage between datasets. As a result, overlap between group education and MNT participants could not be quantified with confidence. Finally, the small number of participants with matched pre/post surveys limited statistical power to detect changes in quality of life, general health, and confidence in problem-solving. Null findings likely reflect limited statistical power rather than program ineffectiveness.

In conclusion, a model for virtual group nutrition education and individual MNT was created and delivered to Older Americans Act clients in Georgia. Effects of the program were evaluated and found to demonstrate feasibility, acceptability, and modest improvement in nutrition knowledge. No detectable effects on participant confidence or health behaviors were demonstrated. These preliminary results highlight the need for ongoing program development and testing of digital interventions this population. Future directions for research include long-term evaluation of virtual nutrition education and MNT on metrics such as nutrition-related knowledge, confidence, and behavior change, as well as ongoing acceptability and feasibility of digital programming. Further, comparison of participant knowledge and satisfaction between individuals who join virtual programming in a congregate setting versus those who log in individually from their home is warranted. Future implementations should consider additional supports to facilitate high survey response rates; these supports include (1) adequate staffing in congregate sites to aid with survey administration, (2) integrating surveys directly into MNT sessions and (3) utilizing incentives for survey completion. With increasing numbers of seniors using internet-connected devices, ample room exists to evaluate the most effective way to utilize these tools in nutrition and health-related information for behavior change. Armed with this information, professionals can expand digital offerings to better meet the needs of their communities, allowing Older American Act clients and others to optimize their nutrition, support their long-term health, and age independently in the place of their choosing.

## Data Availability

The raw data supporting the conclusions of this article will be made available by the authors, without undue reservation.
