# Supplementary material for: Digital nutrition intervention in Older Americans Act programs impacts knowledge and desire to participate in virtual programming
Source: Front Public Health. 2026 Feb 18;14:1695528. doi: 10.3389/fpubh.2026.1695528 (PMC12958140; doi:10.3389/fpubh.2026.1695528)
Supplement: Supplementary file 1 [file Data_Sheet_1.docx]

**Supplementary Table S1. Survey Instruments and Variable Coding**

**A. Confidence & Motivation Items**

| Domain | Item text | Response anchors | Coding for analysis |
| --- | --- | --- | --- |
| Knowledge: Healthy choices | “I have the knowledge to make healthy food choices.” | 1 = Strongly disagree; 5 = Strongly agree | 1 = Agree/Strongly Agree; 0 = otherwise |
| Ability | “I am able to make healthy food choices on my current budget.” | 1 = Strongly disagree; 5 = Strongly agree | 1 = Agree/Strongly Agree; 0 = otherwise |
| Confidence: Shopping | “I feel confident selecting healthy foods in the grocery store.” | 1 = Strongly disagree; 5 = Strongly agree | 1 = Agree/Strongly Agree; 0 = otherwise |
| Confidence: Cooking | “I feel confident cooking & preparing healthy meals.” | 1 = Strongly disagree; 5 = Strongly agree | 1 = Agree/Strongly Agree; 0 = otherwise |
| Motivation | “I feel motivated to make healthy dietary and lifestyle choices.” | 1 = Strongly disagree; 5 = Strongly agree | 1 = Agree/Strongly Agree; 0 = otherwise |
| Sense of control | “I am in control of my own dietary and lifestyle choices.” | 1 = Strongly disagree; 5 = Strongly agree | 1 = Agree/Strongly Agree; 0 = otherwise |

**B. Lifestyle Behavior Items**

| Domain | Unit of assessment | Response | Coding for analysis |
| --- | --- | --- | --- |
| Fruit intake | Servings per day | numeric | 1 = ≥3 servings/day; 0 = <3 |
| Vegetable intake | Servings per day | numeric | 1 = ≥3 servings/day; 0 = <3 |
| Water intake | Cups/day | numeric | 1 = ≥5 cups/day; 0 = <5 |
| Stress relief | Frequency per week | numeric | 1 = ≥3/week; 0 = <3 |

**C. MNT Quality of Life, General Health, and Problem-Solving Confidence Items (assessed at pre and post)**

| Domain | Item text | Response anchors | Coding for analysis |
| --- | --- | --- | --- |
| Quality of life (1-7) | “Taking everything in your life into account, please rate your overall Quality of Life” | 1= Life is distressing, it’s hard to imagine how it could get much worse, 4=Life is so-so, neither good nor bad, 7=Life is great, it’s hard to imagine how it could get much better | 1 = yes, 0 = no |
| General health (1-5) | “Would you say that in general your health is:” | 1= Poor, 5=Excellent | 1 = yes, 0 = no |
| Problem-solving confidence (1-10) | “The next question has to do with how confident you are in your ability to solve problems that you might face in life (for example: I can usually handle whatever comes my way. If I try hard enough, I can overcome difficult problems. I can stick to my aims and accomplish my goals).” | 1=Not at all confident, 10=Very confident |  |

**D. MNT Post-Only Behavior Change Items**

Root question: Which, if any, of the following changes have you made since beginning your nutrition counseling sessions(s)? Check all that apply.

| Domain | Selected choice | Coding for analysis |
| --- | --- | --- |
| Changed intake | “I changed my intake of foods based on my dietitian’s recommendation” | 1 = yes, 0 = no |
| Changed portion sizes | “I changed my portion size based on my dietitian’s recommendation” | 1 = yes, 0 = no |
| Changed meals made at home | “I increased the number of meals I make at home” | 1 = yes, 0 = no |
| Changed physical activity | “I’ve added more physical activity” | 1 = yes, 0 = no |
| Changed stress relief activity | I’ve added mindful practices to aid with stress relief (meditation, reading, etc.) | 1 = yes, 0 = no |
| Changed Other | “Other” | 1 = yes, 0 = no |

**Supplementary Table 2.** Session-level paired knowledge score changes pre to post-assessment

| Session | n (paired) | Mean Diff | SD Diff | SE Diff | t | 95% CI (Diff) | p-value | Mean Diff (pct pts) | 95% CI (pct pts) | Cohen’s dz |
| --- | --- | --- | --- | --- | --- | --- | --- | --- | --- | --- |
| 1 | 206 | 0.024 | 0.220 | 0.015 | 1.97 | −0.006 to 0.055 | 0.115 | 2.43 | −0.60 to 5.45 | 0.11 |
| 2 | 237 | 0.047 | 0.196 | 0.013 | 1.97 | 0.022 to 0.072 | 0.00029 | 4.68 | 2.18 to 7.19 | 0.24 |
| 3 | 191 | 0.030 | 0.182 | 0.013 | 1.97 | 0.004 to 0.056 | 0.024 | 3.00 | 0.40 to 5.60 | 0.16 |
| 4 | 165 | 0.007 | 0.132 | 0.010 | 1.97 | −0.013 to 0.028 | 0.488 | 0.72 | −1.32 to 2.75 | 0.05 |
| 5 | 199 | 0.015 | 0.159 | 0.011 | 1.97 | −0.007 to 0.037 | 0.178 | 1.52 | −0.70 to 3.75 | 0.10 |
| 6 | 196 | 0.044 | 0.248 | 0.018 | 1.97 | 0.009 to 0.079 | 0.014 | 4.39 | 0.89 to 7.88 | 0.18 |
| 7 | 235 | −0.012 | 0.165 | 0.011 | 1.97 | −0.033 to 0.009 | 0.252 | −1.23 | −3.35 to 0.88 | −0.07 |
| 8 | 234 | 0.007 | 0.126 | 0.008 | 1.97 | −0.009 to 0.024 | 0.375 | 0.73 | −0.89 to 2.36 | 0.06 |
| 9 | 231 | 0.001 | 0.179 | 0.012 | 1.97 | −0.023 to 0.024 | 0.961 | 0.06 | −2.26 to 2.38 | 0.00 |
| 10 | 215 | 0.00008 | 0.159 | 0.011 | 1.97 | −0.021 to 0.021 | 0.994 | 0.01 | −2.13 to 2.15 | 0.00 |
| 11 | 195 | 0.014 | 0.161 | 0.012 | 1.97 | −0.008 to 0.037 | 0.215 | 1.44 | −0.84 to 3.71 | 0.09 |
| 12 | 190 | 0.025 | 0.185 | 0.013 | 1.97 | −0.002 to 0.051 | 0.069 | 2.46 | −0.19 to 5.10 | 0.13 |
| 13 | 143 | 0.007 | 0.205 | 0.017 | 1.98 | −0.027 to 0.041 | 0.699 | 0.66 | −2.73 to 4.05 | 0.03 |
| 14 | 110 | 0.029 | 0.209 | 0.020 | 1.98 | −0.010 to 0.069 | 0.145 | 2.92 | −1.02 to 6.87 | 0.14 |
| 15 | 60 | 0.006 | 0.174 | 0.022 | 2.00 | −0.039 to 0.051 | 0.806 | 0.56 | −3.94 to 5.06 | 0.03 |
| 16 | 56 | −0.023 | 0.140 | 0.019 | 2.00 | −0.061 to 0.014 | 0.221 | −2.32 | −6.08 to 1.44 | −0.17 |
| 17 | 59 | 0.015 | 0.163 | 0.021 | 2.00 | −0.028 to 0.058 | 0.484 | 1.50 | −2.76 to 5.75 | 0.09 |
| 18 | 66 | 0.013 | 0.174 | 0.021 | 2.00 | −0.030 to 0.056 | 0.543 | 1.31 | −2.97 to 5.60 | 0.08 |
| 19 | 46 | 0.043 | 0.171 | 0.025 | 2.01 | −0.008 to 0.094 | 0.097 | 4.28 | −0.80 to 9.35 | 0.25 |
| 20 | 53 | −0.027 | 0.144 | 0.020 | 2.01 | −0.066 to 0.013 | 0.183 | −2.67 | −6.65 to 1.30 | −0.19 |

**Supplementary Table 3**. Kuder-Richardson 20 coefficient ( KR-20) assessment of knowledge session assessments 1-20

| Session | N pre-session assessment | N post-session assessment | Kr20 pre | Kr20 post |
| --- | --- | --- | --- | --- |
| 1 | 179 | 182 | 0.38 | 0.38 |
| 2 | 216 | 192 | 0.22 | 0.35 |
| 3 | 171 | 165 | 0.33 | 0.35 |
| 4 | 149 | 153 | 0.55 | 0.54 |
| 5 | 167 | 166 | 0.45 | 0.56 |
| 6 | 141 | 120 | 0.33 | 0.33 |
| 7 | 220 | 217 | 0.41 | 0.48 |
| 8 | 225 | 211 | 0.11 | 0.13 |
| 9 | 168 | 168 | 0.47 | 0.55 |
| 10 | 204 | 172 | 0.43 | 0.40 |
| 11 | 172 | 170 | 0.58 | 0.55 |
| 12 | 169 | 150 | 0.46 | 0.58 |
| 13 | 116 | 111 | 0.50 | 0.55 |
| 14 | 80 | 68 | 0.38 | 0.59 |
| 15 | 57 | 50 | 0.70 | 0.80 |
| 16 | 43 | 46 | 0.46 | 0.74 |
| 17 | 31 | 28 | 0.21 | 0.06 |
| 18 | 57 | 50 | 0.32 | 0.58 |
| 19 | 34 | 36 | 0.78 | 0.69 |
| 20 | 47 | 39 | 0.30 | 0.17 |
