# Supplementary figures and images for: Digital nutrition intervention in Older Americans Act programs impacts knowledge and desire to participate in virtual programming
Source: Front Public Health. 2026 Feb 18;14:1695528. doi: 10.3389/fpubh.2026.1695528 (PMC12958140; doi:10.3389/fpubh.2026.1695528)

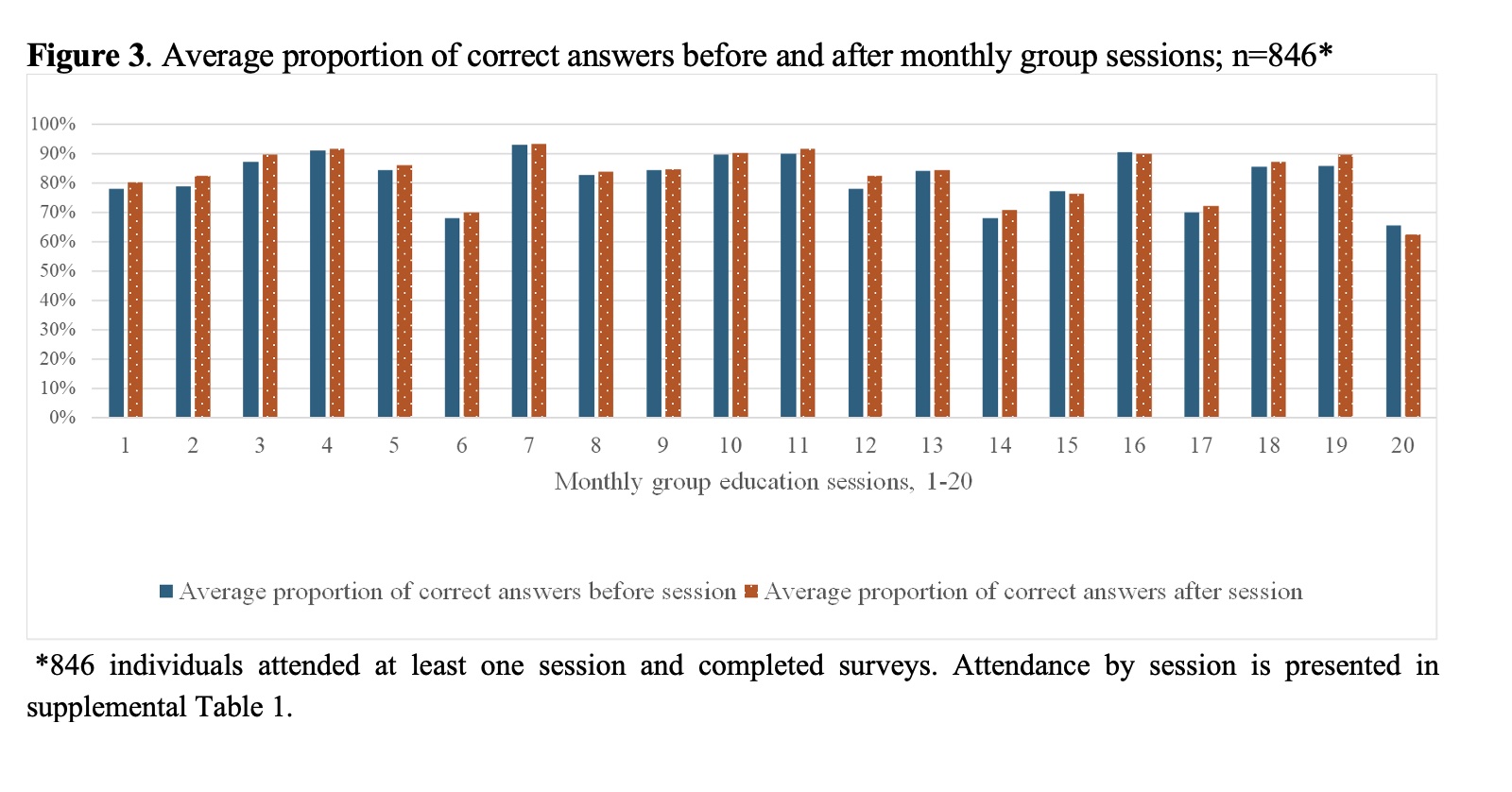

Supplement: Supplementary file 2 [file Image_3.jpeg]

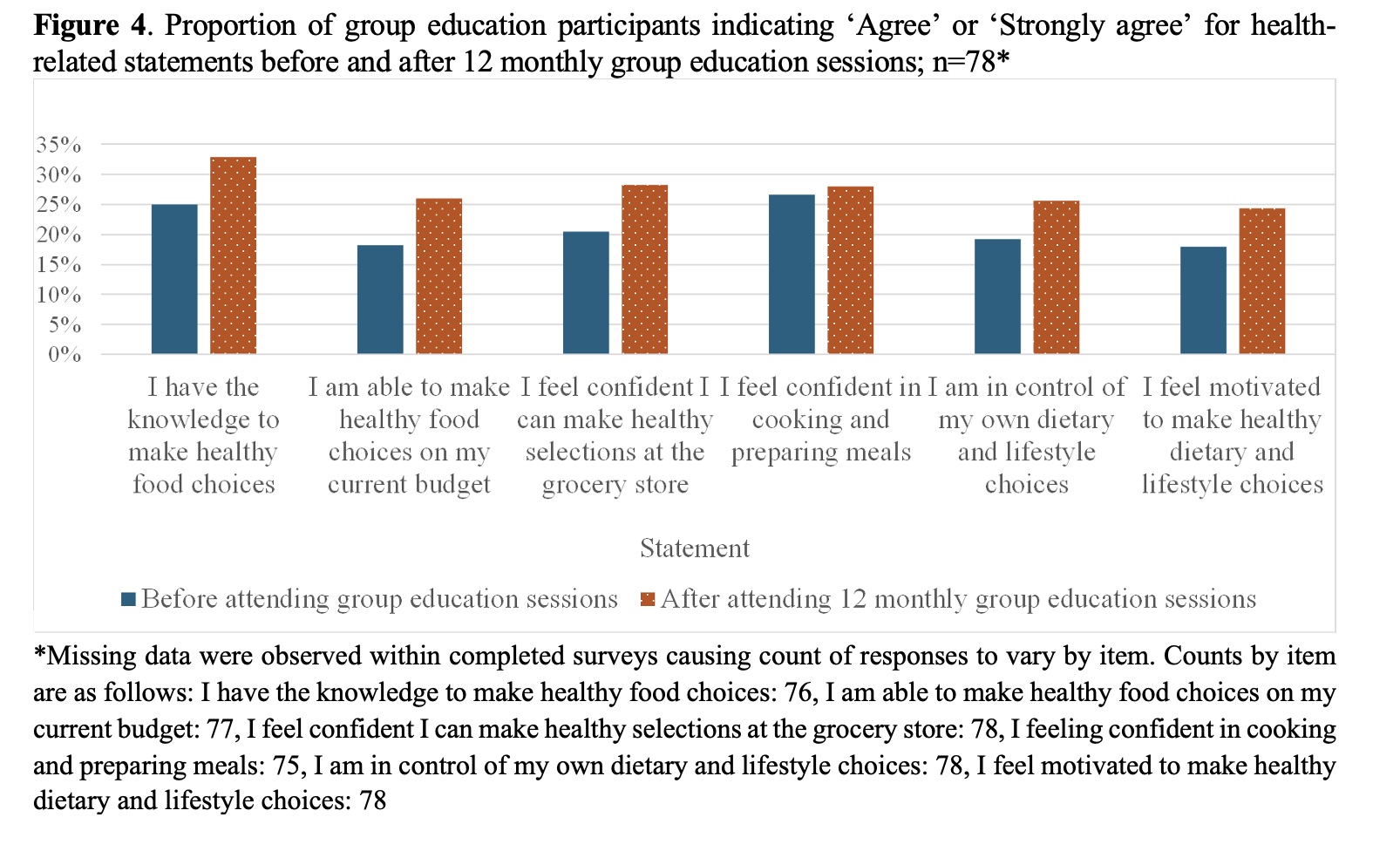

Supplement: Supplementary file 3 [file Image_4.jpeg]

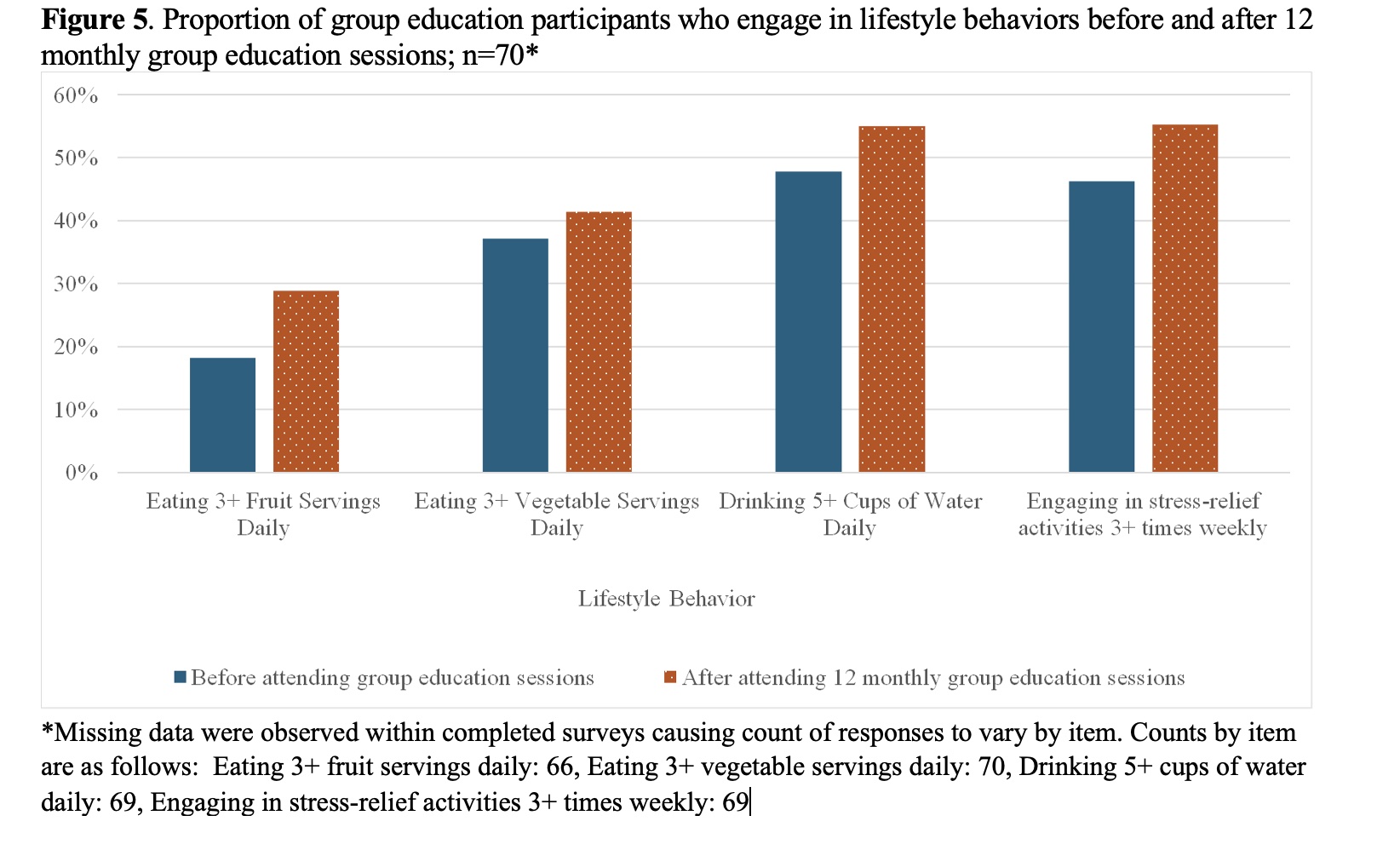

Supplement: Supplementary file 4 [file Image_5.jpeg]

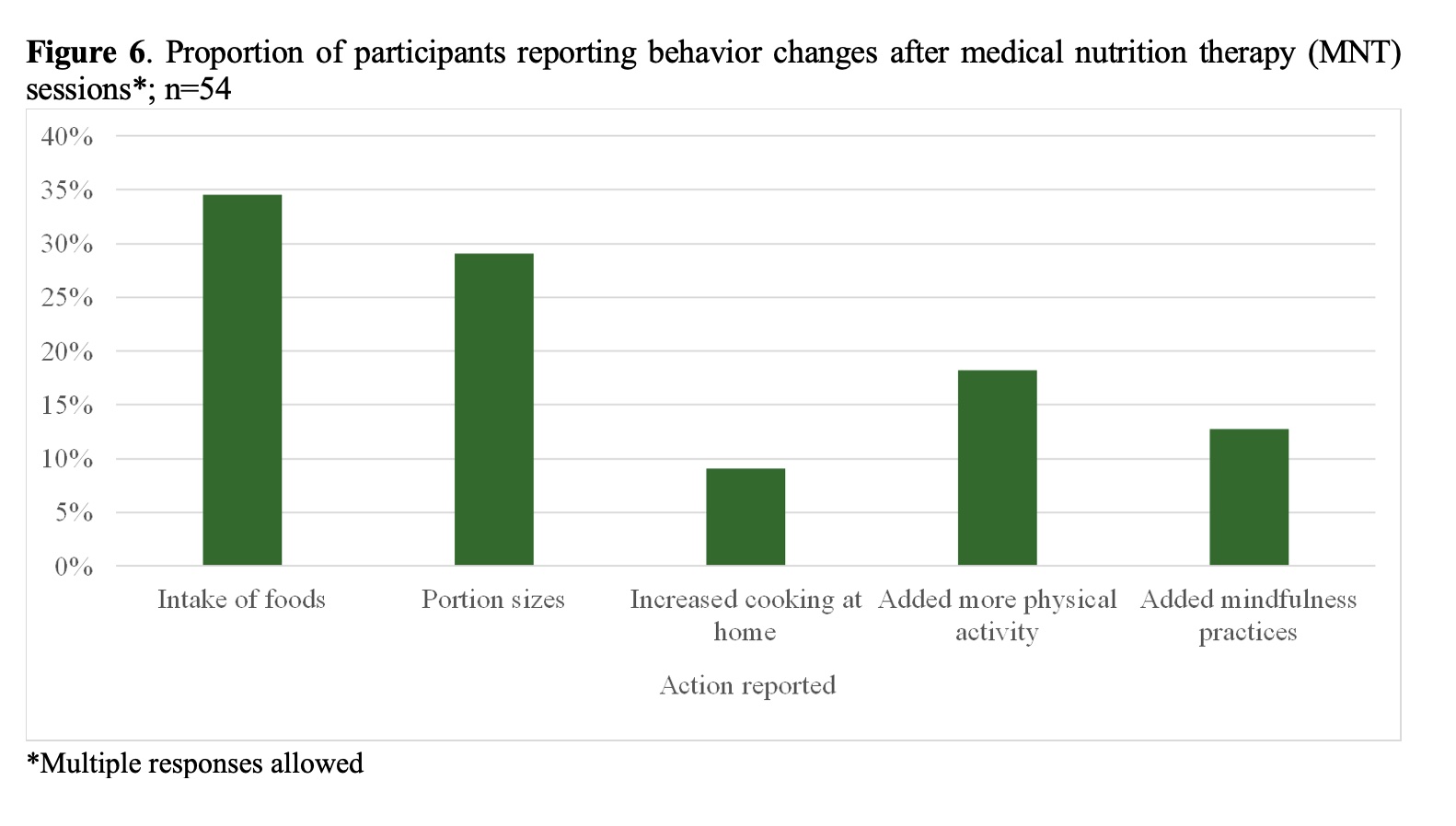

Supplement: Supplementary file 5 [file Image_6.jpeg]
